# Editorial: Neuronal Polarity: Establishment and Maintenance

**DOI:** 10.3389/fncel.2018.00137

**Published:** 2018-05-23

**Authors:** Froylan Calderon de Anda, Annette Gaertner

**Affiliations:** ^1^Center for Molecular Neurobiology Hamburg, Research Group Neuronal Development, University Medical Center Hamburg-Eppendorf, Hamburg, Germany; ^2^Department of Development and Regeneration, Faculty of Medicine, KU Leuven, Leuven, Belgium

**Keywords:** neuronal polarization, neuronal migration disorders, axon, dendrites, neuronal differentiation

The term neuronal polarization denotes cellular compartmentalization, which leads to an asymmetric cellular morphology. This compartmentalization is depicted by the morphological and functional separation of axons and dendrites. The axon transmits signals over long distances while dendrites are shorter and receive information. One fundamental question in neurobiology is how neurons form one axon and several dendrites. This issue has been tackled for several years using neurons growing in culture. However, neurons develop in a complex and changing environment that is not easy to reproduce *in vitro*; thus, in the recent years, different animal models and organotypic cultures were used to investigate neuronal differentiation *in situ*. This research topic on the establishment and maintenance of neuronal polarity brings together important scientific contributions that help to better understand several aspects of neuronal development in vertebrates.

## Neuronal polarization *in situ*: from genesis to migration

Studying neuronal development *in situ* is instrumental to understand how external cues help to sculpt neuronal morphology during polarization. This issue is analyzed in reviews and original research articles on this topic. With a global perspective, Hansen et al. provided an extensive overview of the extrinsic and intrinsic molecular mechanisms currently associated with neuronal differentiation in the developing cortex. In the developing cortex, the differentiation and development of neurons start with neuronal progenitors. Arai and Taverna summarized the current knowledge regarding cellular polarity in neuronal progenitors. The intracellular polarized architecture of neuronal progenitors dictates their behavior and thus the type of cellular divisions they undergo. This ultimately might determine the number and type of neurons produced, and the organization of the inherited cytoplasm to their progeny. Ong and Solecki reviewed the latest results, which support the role of seven in absentia homolog (Siah) ubiquitin ligases controlling the onset of neuronal polarization in the developing cerebellum. Siah is involved in post-translational regulation of cellular polarity and regulates neuron migration through cellular adhesion and the cytoskeleton. Moreover, the authors speculate on additional targets and interactors found in non-neuronal cells.

## Neuronal migration and axon formation: the transition toward the final polarization

In the developing cortex, newly formed neurons migrate to their final destination to eventually find their location where they will form connections. Importantly, neuronal polarization occurs while neurons migrate (Noctor et al., 2004; de Anda et al., 2010; Namba et al., 2014; Sakakibara et al., 2014). Kon et al. analyzed different cellular, morphological changes and underlying molecular mechanisms during initial steps of neuronal polarization. The review by Frotscher et al. summarized the role of Reelin signaling during neuronal migration in the developing cortex. Reelin is important for the orientation and maintenance of the leading process of migrating neurons by phosphorylating and inactivation of Cofilin, an actin-binding protein.

Gorelik et al. exemplified how molecules such as *Serping1* (C1 inhibitor, a member of the complement cascade), known for very distinct biological functions, are “hijacked” for regulating cortical development. Knockdown or knockout of *Serping1* delayed migration of neurons in mice suggesting it's importance in cortical development at several stages of neuronal differentiation. Similarly, Laguesse et al. identified a molecular pathway, which regulates the acetylation of Connexin-43 (Cx43), that might modulate neuronal motility in the developing cortex. Elp3 acetylates Cx43 while histones deacetylase 6 (HDAC6) controls levels of acetylated Cx43.

Finally, during migration neurons initiate axon formation—a morphological landmark of neuronal polarization. Lee et al. investigated the role of Calsyntenin-1 (Clstn-1), which is a kinesin adaptor, in organizing microtubule polarity during axon development. Using a zebrafish model and high-speed *in vivo* imaging, Lee et al. imaged sensory neurons during axon formation and branching. Their results suggest an important role for Clstn-1 in the regulation of microtubule polarity in actively branching axons.

## Dendrite formation: the final step before connectivity

Once neurons arrive at their final destination they form dendrites, spines, and establish connections. In addition, the compartmentalization of axon and dendrites is defined at the subcellular level. At this stage, the selective sorting into axons and dendrites becomes crucial for differentiating their distinct function (Zollinger et al., 2015). A sorting barrier, the axon initial segment (AIS), assembled at this developmental time point (Zollinger et al., 2015), and the selective recruitment of cargo by different motor proteins supports the polarized transport (Rasband, 2010). Janssen et al. illustrated the involvement of myosin-V, a dendritic transporter, in the clustering of kinesin-driven cargoes at the AIS, precisely in actin-rich hotspots. The cargo retrieval from these hotspots is taken over by a yet unidentified protein. To understand how neurite formation takes place, Wang et al. investigated the role of microRNA-189 (miR-182) in neurite outgrowth. Interestingly, they found that miR-182 plays a role in axon and dendrite extension through a molecular pathway, which involves AKT phosphorylation and inhibition of BCAT2. With a cellular and mathematical approach, Tomba et al. analyzed the contribution of actin-rich structures (actin waves), which travel along the neurite shaft from the cell body to the neurite tip (Ruthel and Banker, 1999; Winans et al., 2016; Mortal et al., 2017), in supporting neurite outgrowth. Their mathematical modeling leads to a quantitative characterization of the generation of actin waves. In dendrites, actin was mainly described to be important for spine morphology and dynamics. Konietzny et al. provided an interesting overview of recent findings showing the role of dendritic actin in dendrite morphology and protein traffic and the regulation of dendritic actin dynamics. Finally, once neurons initiate building networks, the specialization of subcellular domains allow for proper connections. The insertion of ion channels in proper places ensures adequate propagation of electrical impulses—a crucial step in the development of a fully differentiated neuron. Duménieu et al. reviewed recent data on the organization and mechanisms behind the segregation of voltage-gated Potassium and Sodium channels in dendrites.

Our knowledge regarding neuronal polarization and maintenance *in situ* is limited. However, in recent years we have advanced our understanding of this matter. This research topic reunites studies to emphasize the importance of studying neuronal differentiation *in situ*. Overall, it helps to better understand the cellular and molecular mechanism underlying neuronal polarization in a complex environment, different from the culture conditions. It is our hope that this knowledge will pave the way for future contributions in the field.

## Author contributions

FCdA and AG wrote the Editorial and coordinated the Topic.

### Conflict of interest statement

The authors declare that the research was conducted in the absence of any commercial or financial relationships that could be construed as a potential conflict of interest.
